# Factor structure and internal reliability of breast cancer screening Champion’s Health Belief Model Scale in Yemeni women in Malaysia: a cross-sectional study

**DOI:** 10.1186/s12905-021-01543-7

**Published:** 2021-12-29

**Authors:** Sarah Noman, Hayati Kadir Shahar, Hejar Abdul Rahman, Suriani Ismail, Musheer A. Aljaberi, Muzaphar N. Abdulrahman

**Affiliations:** 1 Department of Community Health, Faculty of Medicine and Health Sciences, Universiti Putra Malaysia, 43400 Serdang, Malaysia; 2 Faculty of Medicine and Health Sciences, Taiz University, Taiz, Yemen; 3 Malaysian Research Institute of Ageing (MyAgeing), 43400 Serdang, Malaysia

**Keywords:** Exploratory factor analysis, Champion’s Health Belief Model Scale, Breast cancer, Breast cancer screening, Yemeni women

## Abstract

**Background:**

The reliability and validity of the Champion’s Health Belief Model Scale **(**CHBMS) used in assessing the belief of women regarding breast cancer (BC) and breast cancer screening (BCS) have been examined on various populations. However, the use of this tool has not been adequately assessed for its validity in ethnic minorities. This study assessed the validity and reliability of CHBMS by analyzing the factor structure and internal reliability of the factors among Yemeni women in Malaysia.

**Methods:**

A survey was conducted among 103 female teachers from 10 schools. SPSS version 22.0 was utilized in analyzing the data. Descriptive statistics were computed for the socio-demographic characteristics. The Cronbach’s alpha coefficients were used in assessing the internal reliability. The Exploratory Factor Analysis (EFA) was used to analyze the factor structure of the translated items. Parallel analysis was performed to determine the number of factors accurately.

**Results:**

The alpha coefficients of the factors had acceptable values ranging between 0.76 and 0.87. The factor analysis yielded six and five factors for breast self-examination (BSE) and mammography (MMG), with a total explained variance of 47.69% and 52.63%, respectively. The Kaiser–Meyer–Olkin (KMO) index values of 0.64 and 0.72, and the Bartlett’s Test of Sphericity (*P* = 0.0001) for BSE and MMG, respectively, verified the normality distribution and the adequacy of the sample size for EFA. All the items on each factor were from the same construct that were consistent with the number of factors obtained in the scale development study. The items achieved adequate factor loadings that ranged between 0.47 and 0.88.

**Conclusions:**

The translated version of the CHBMS is a validated scale used in assessing the beliefs related to BC and BCS among Yemeni women living in Malaysia. Healthcare workers could use the scales to assess women’s beliefs on BC and BCS. This instrument could be used to test the effectiveness of the intervention programs.

## Background

Breast cancer (BC) is the most common type of cancer detected. However, it remains the leading cause of cancer mortality among women worldwide [1]. Although the prevalence of BC is lesser in developing countries than the developed countries [2], the mortality rate is higher in the former [3]. The significant contrast in BC mortality could be attributed to the lower frequency of breast cancer screening (BCS) in the developing countries [4], which would increase the rate of survival and increase the possibility for early detection and successful treatment of BC [5]. Therefore, Yemen, as a developing country, is a case in point, with a low rate of BCS practice among its women, ranging between 11 and 17.4% [6, 7], therefore resulting in a higher probability for a later stage of identification of most BC cases [8]. Influenced by their status as foreign immigrants, Yemeni women in Malaysia are significantly impacted by the challenges and barriers in accessing BCS-related information and services available for Malaysian women, thus resulting in the low level of BCS uptake among this ethnic group [9, 10].

BCS behavior may be influenced by a multitude of factors. The causal relationship between institutional factors, patients, and providers with disparities in healthcare treatment among racial and ethnic groups has been identified by the Panel on Racial and Ethnic Disparities in Medical Care in its report in 2003 [11]. The differences in medical care are significantly attributed to cultural beliefs about medical healthcare [12]. Besides that, the health beliefs influence the women’s BCS behavior [13, 14]. The explanation for this behavior has been made in studies performed in relation to a variety of theories. The description of the meaning of BC and BCS in such studies was made on the relation of one concept to one behavior or through a more compound framework. At present, the theories most commonly used in promoting BCS are the Social Cognitive Theory (SCT), the Theory of Planned Behavior (TPB), the Transtheoretical Model (TTM), the Ecological Model (EM), the Health Promotion Model (HPM), and the Health Belief Model (HBM). HBM has been used successfully in numerous attempts to customize the interventions to women's health beliefs in increasing BCS in several populations, and the model’s variables have been reported to predict BCS behaviors in most of these populations [15–27].

Although evaluations on the reliability and validity of the standardized HBM on BCS-associated women’s health beliefs were performed [12, 28–39], the tool's validity for use in ethnic minorities has not been adequately assessed [13]. Thus, further investigation is required in comprehending the best method in measuring women’s health beliefs in culturally diversified populations. It was evident that using tools containing items with biased culture could yield invalid decisions on the efficacy of interventions [40]. Additionally, based on such biased conclusions, the consequent implementation of initiatives and interventions is ineffectual for specific ethnic or racial groups [12]. Therefore, this study aims to evaluate the Champion’s Health Belief Model Scale’ (CHBMS) validity and reliability by analyzing the factor structure and internal reliability of the factors that may affect the practice of BCS among Yemeni women in Malaysia.

## History of the health belief model

Initially, the development of the HBM was undertaken in the 1950s by social psychologists of the U.S. Public Health Service in explaining the prevalence of the people’s failure to participate in preventive disease programs. At that point, there were concerns among the researchers and health professionals over the small number of people getting screened for tuberculosis, although the mobile X-ray would be brought to the people’s neighborhood [41–43]. Primarily, Hochbaum [41] reviewed the perception regarding the people’s belief about their susceptibility to tuberculosis disease and their beliefs about the benefits of early detection. Their belief influences the likelihood of early intervention on the susceptibility to a condition, opinion on possible serious sequelae, belief that the measures would minimize either the susceptibility or severity of the condition and understanding that the benefits outweigh the risks.

Later on, the extension was made to the model to study people's responses to symptoms [44] and their actions in response to a diagnosed disease, particularly adherence to medical regimens [45]. The dimension was reformulated to fit the medically established cases (instead of only risk reduction), consisting of the susceptibility factors, taking of the diagnosis, and overall susceptibility to the disease [46]. The HBM includes a number of concepts that speculate the reason behind an individual’s decision to take action to screen, prevent, or control diseases. These concepts involve self-efficacy, susceptibility, cues for action, benefits and barriers to a specific behavior, and seriousness. Besides, as suggested by the model, health-related behavior could be indirectly affected by perception that is influenced by knowledge and diverse demographic factors [47].

### Application of the health belief model scales for breast cancer screening

The evaluation of BCS-related constructing the HBM was linked to the behaviors toward both mammography (MMG) screening and breast self-examination (BSE). Initially, the development and validation of the BSE scale were performed by Champion in 1984. Later on, in 1993, a revision was made to this scale [48]. The benefits and barriers of MMG were then revised [49]. CHBMS emphasized that health behavior is affected by threats from health problems. There is a higher probability for women to seek screening tests if the susceptibility to the risk of BC is considered or believe that the disease is a serious matter. Women with high health motivation who believe that the benefits of screening examination outweigh the barriers, and also those who are capable of successfully performing a behavior have a higher probability of undertaking a screening test [48, 49].

## Methods

### Design

The present research is a cross‐sectional study aims to measure the validity and reliability of the CHBMS.

### Settings

This study involves a sample of Yemeni women living in the Klang Valley, Malaysia, between October 2016 to March 2017. The inclusion criteria are as follows: (1) Yemeni female teachers teaching at Arabic schools located in the Klang Valley area; (2) teachers who agree to participate in the study by signing the consent form; 3. aged 20 years and above. The exclusion criteria for the participants are: (1) pregnant or lactating teachers; (2) teachers who reach retirement age during the study; (3) teachers who have been diagnosed with BC. The participation of a total of 168 all-female teachers from 10 schools were sought after, and 120 gave their consent in participating and were found eligible. The sample size of the current study is sufficient for the exploratory factor analysis (EFA) to be performed due to the existence of several factors and several items for each factor, and due to the communalities range of the items that were between moderate and high [50].

### Study instruments

The following subscales have been included in the instrument for the purpose of data collection of the study;

1. Demographic information for the assessment of variables namely age, marital status, income, familiarity with BCS, and family history of BC.
2. The CHBMS for BCS in measuring the health beliefs of the participants towards BC, and towards its screening tests [48, 49]. The scale comprises of 53 items, and uses a 5-point Likert scale based on the following coding: Strongly disagree (1 point), disagree (2 points), neutral (3 points), agree (4 points), and strongly agree (5 points). The CHBMS comprises of eight subscales: (a) perceived susceptibility (5 items), (b) perceived seriousness (7 items), (c) benefits‐BSE (6 items), (d) barriers‐BSE (6 items), (e) perceived confidence (11 items), (f) health motivation (7 items), (g) benefits‐MMG (6 items), and (h) barriers MMG (5 items). The scores were then summated for analysis. Greater feelings in relation to the constructs are reflected by the higher scores [49]. Except for barriers that negatively associate screening behaviors, all other scales positively correlate with screening behaviors.

Back-translation is the preferred translation technique that utilizes a panel of interpreters and experts to translate the items from the source to the target language, and then in back-translating, the items again into the source language [51, 52]. Once an agreement on the meaning and word choice for the items is reached, and upon the confirmation that the tool conveys clear understanding to the target population, a test must be carried out on a small group of participants. The investigators should utilize words that favor and are commonly used by the target population to ensure the cultural suitability of the translated tool for the participants [53].

Hence, the translation of the questionnaire was made based on the World Health Organization’s professional translation process [54]. The service of two independent health professionals, who are Arabic native speakers and fluent in English and familiar with the terminology of the research topic, was sought after in administering the forward translation of the original questionnaire from English to the Arabic language. Then two translators who are bilingual in English and Arabic reviewed the translated questionnaire. Then, the study researchers performed another review of the translated version of the questionnaire, and a comparison was made with the original version. Based on the comparison, the accuracy of the first draft of the questionnaire was decided and agreed upon. The questionnaire was then translated back into English by an independent translator based on the recommendations for instrument adaptation. The translator’s mother tongue is English, with no prior knowledge of the questionnaire [54]. The result of the back-translation showed that no change in wordings was required since the back-translation produced a translation that was almost identical to and matched the original meaning of the English version.

Next, seven professional expert panelists' service was sought to examine and assess the questionnaire’s face validity and content validity index (CVI) [55]. The panel of professional experts comprised of three medical doctors, a consultant radiologist specializing in screening and diagnosis of BC, a professor in psychology, and two nursing faculty members. The feedback from the panelists were mainly on the simplicity and understandable content. Changes were later made on relevant items based on the suggestions by the professional experts. Upon the professional judges’ examination and assessment of the revised tool, the questionnaire was thus determined to be culturally appropriate with an indication of a good CVI (CVI = 0.95%). The translated questionnaire was then administered for a pre-test to 30 Yemeni female schoolteachers who were not included in this study. The pre-test respondents were asked about any words or expressions they found difficult to understand or anything inappropriate. Accordingly, some words and phrases were improved, or the alternatives given for a change, and the final version of the questionnaire was then developed.

### Statistical analysis

The Statistical Package for Social Sciences version 22.0 was used for the data analysis. The computation of descriptive statistics was made for socio-demographic characteristics. Reliability was assessed using Cronbach’s alpha coefficients, while internal consistency was examined on the items for each subscale. The alpha levels of ≥ 0.70 are considered desirable.

The EFA was used to analyze the factor structure of the translated items. Parallel analysis was performed to determine the number of factors accurately. As the most widely used method in factor analysis, principal axis factoring was utilized in extracting the factors. Furthermore, when the results that emerged from the principal component analysis were compared to the principal axis factoring, the results were found to be reliable [57–59], although the results produced were somehow similar. Since more variance were included, the principal component analysis solution had more items with cross-loadings, thus giving the implication that an item’s variance could be explained by multiple factors [56].

The Kaiser–Mayer–Olkin (KMO) was used to examine the sampling adequacy measure [57]. The KMO test value varies between 0 and 1, and a higher value means a more suitable analysis. According to Tabachnick and Fidell [58], KMO should be equal to or higher than 0.60 in order to continue with factor analysis. In more detail, Hair, Black [59] pointed out that the KMO values with 0.90 s are excellent; 0.80 s are good, 0.70 s are middling; 0.60 s are mediocre; 0.50 s are acceptable but miserable; and below 0.50 is unacceptable. The Bartlett’s Test for Sphericity (*P* < 0.05) was applied to confirm that the items have patterned relationships [60]. The factors obtained were orthogonally rotated using the varimax method since it provided a better and simpler structure [56]. Besides that, the factors were assumed to be independent [61]. Furthermore, when oblique rotation was run to examine factor correlation, the results showed correlation values below 0.3, suggesting low correlation among the factors. Hence, orthogonal rotation is recommended [61]. Based on Stevens [62], factor loadings for a sample size of 100 are significant at the level of 0.01 when they are > 0.512 was therefore applied.

One of the most significant concerns and the most challenging and critical stages of factor analysis is deciding the number of factors [63]. The most widely used approach to determine number of factors are eigenvalue greater than 1 and the examination of the scree plot. Another approach used in determining the number of factors is Parallel Analysis as suggested by Horn [64]. Parallel analysis is a consistent and acceptable method used to precisely decide the number of factors [63]. To determine the number of factors, this method utilizes random data simulation. Besides generating the actual (real) data set, the parallel analysis approach generates a random simulative (artificial) data set using the Monte Carlo Simulation Technique and then calculates the estimated eigenvalues. In this method, the number of factors is considered significant when the eigenvalue in the simulative sample is higher than that of the actual data [65]. Therefore, to accurately ascertain the number of factors, parallel analysis has been used in this study. Finally, the analysis was re-performed with the obtained number of factors as suggested by parallel analysis.

## Results

A total of 120 questionnaires were initially distributed, and only103 of them were completed with a response rate of 86%. Statistical analyses were conducted on the 103 completed and returned questionnaires. The results of the socio-demographic characteristics show that 79.5% of the respondents were married, and 14.6% of them were single, with a mean age of 33.99 (SD = 6.49) years. The mean income is RM 1798.06 (SD = 484.87). BC family history was reported by 11.7% of the respondents. Most of the respondents have read or heard about BCS (83.5%) (Table 1). Further information regarding the participants’ demographic characteristics is presented in Table 1.

**Table 1:** Characteristics of socio-demographic of the respondents

Variables	Mean ± SD	Frequency “N”	Percentage “%”
Age (years) (min–max)	33.99 ± 6.49 (23–50)		
Income (RM) (min–max)	1798.06 ± 484.87 (800–3500)		
Marital status			
Married		82	79.5
Divorced/Separated		5	4.9
Widowed		1	1.0
Single		15	14.6
Family History of BC			
Yes		12	11.7
No		91	88.3
Read/heard about BCS			
Yes		86	83.5
No		17	16.5

*SD* standard deviation

### Internal reliability

The calculation of each factor’s internal consistency reliability was made using Cronbach’s coefficient alpha. The factor of “susceptibility” obtained an internal reliability of α = 0.79; the factor of “seriousness” α = 0.83; the factor of “benefits of BSE” α = 0.76; the factor of “barriers of BSE” α = 0.81; the factor of “confidence on performing BSE” α = 0.87; the factor of “health motivation” α = 0.86; the factor of “benefits of MMG” α = 0.87; and the factor of “barriers of MMG” α = 0.87. Table 2 shows the summary results of the internal consistency reliability for CHBMS.

**Table 2:** Reliability of the dimensions of the HBMS for breast self-examination and mammography

Factor	Number of items	Mean ± SD	Cronbach’s alpha
SUS	5	18.78 ± 3.44	0.79
SER	7	26.13 ± 5.46	0.83
BEN (BSE)	6	23.17 ± 3.35	0.76
BAR (BSE)	6	13.18 ± 4.21	0.81
CON	11	32.03 ± 6.47	0.87
HM	7	28.10 ± 5.13	0.86
BEN (MMG)	6	21.74 ± 3.85	0.87
BAR (MMG)	5	14.59 ± 4.02	0.87

*SUS* susceptibility, *SER* seriousness, *BEN* benefits, *BAR* barriers, *CON* confidence, *HM* Health motivation, *SD* standard deviation

### Exploratory factor analysis

#### Breast self-examination (BSE)

To determine if the data is appropriate for EFA, the screening of the 42 items related to BSE was conducted prior to the analysis. The examination of the correlation matrix indicated that there are patterned relationships amongst the variables. Besides that, screening revealed an absence of multicollinearity. The observation of Bartlett’s Test of Sphericity (*P* = 0.0001) confirmed of the existence of patterned relationships among the items. Finally, the examination of KMO (0.64) demonstrated the adequacy of the sample size for EFA. To this end, it is demonstrated by the preliminary findings that factor analysis could be performed on the subscales related to BSE.

The initial examination of the eigenvalue displayed 12 factors to be extracted, with a total explained variance of 62.84. However, the use of the scree test inspection revealed a clear break after the sixth factor. As mentioned earlier, further evidence for the proposed use of parallel analysis is provided for an easier decision on the number of factors. Hence, parallel analysis was used in this study to achieve this purpose. The examination of Table 3 clearly shows that the eigenvalue of the first factor in the actual data is 6.35, whereas in the simulative data set, it is 2.69. The eigenvalue of the second factor in the actual data is 4.58, while in the simulative data, it is 2.43. The eigenvalue of the third factor in the actual data is 4.18, while in the simulative data, it is 2.27. The eigenvalue of the fourth factor in the actual data is 3.31, while in the simulative data, it is 2.14. The eigenvalue of the fifth factor in the actual data is 2.36, whereas, in the simulative data, it is 2.03. The eigenvalue of the sixth factor in the actual data is 2.27, while in the simulative data, it is 1.92. The case concerning the move from the sixth to the seventh factor is different. Accordingly, the number of the scale factors is determined to be limited to six since the eigenvalue of the simulative data of the seventh factor is higher than that of the actual data. The eigenvalue of the seventh factor in the actual data is 1.62, while in the simulative data, it is 1.82. This situation must be regarded as the point where the decision about the number of factors is introduced by parallel analysis.

**Table 3:** Eigen values of the actual data and the simulative data

Factor	Eigenvalues of the actual data	Eigenvalues of the simulative data
1	6.35	2.69
2	4.58	2.43
3	4.18	2.27
4	3.31	2.14
5	2.36	2.03
6	2.27	1.92
7	1.62	1.82

There is no correspondence between the number of factors obtained from the eigenvalue approach and those obtained from the original scale developmental study [48]. However, there is correspondence between the number of factors obtained through the parallel analysis and scree plot approaches and those obtained from the original scale development study.

Based on the explanation given above, a decision was made for the number of factors in the current study to be six, and the re-run was performed on the analysis of the actual data, with the restricted number of factors. The total explained variance due to the re-performed EFA with a limited number of factors to six is 47.69%.

The examination of Table 4 demonstrates that by using the above-mentioned criteria, the EFA has achieved a simple structure in which each factor is represented by several items that each load strongly only on that factor. Each factor is sufficiently identified to contain at least three to five items with significant loadings suggesting a stable and solid factor [64]. The first factor is made up of confidence items; the second factor is made up of health motivation items; the third factor is made up of seriousness items; the fourth factor is made up of barriers (BSE) items; the fifth factor is made up of susceptibility items, and the sixth factor is made up of benefits (BSE) items. It is also clear that the range of the rotated factor loadings in the first factor is between 0.48 and 0.86; the range in the second factor is between 0.48 and 0.87; the range in the third factor is between 0.55 and 0.75; the range in the fourth factor is between 0.56 and 0.72; the range in the fifth factor is between 0.52 and 0.73, and the range of the sixth factor is between 0.47 and 0.70. It is therefore concluded that the results of the exploratory factor analysis for subscales related to BSE are consistent with those obtained in the original scale developmental study conducted by Champion [48].

**Table 4:** Factor analysis results of CHBMS for BSE

Factor 1		Factor 2		Factor 3		Factor 4		Factor 5		Factor 6	
Item	Factor loading	Item	Factor loading	Item	Factor loading	Item	Factor loading	Item	Factor loading	Item	Factor loading
CON 8	0.86	HM 2	0.87	SER 4	0.75	BAR 3	0.72	SUS 3	0.73	BEN 4	0.70
CON 2	0.75	HM 3	0.87	SER 3	0.72	BAR 5	0.68	SUS 2	0.72	BEN 3	0.58
CON 11	0.67	HM 4	0.81	SER 1	0.68	BAR 1	0.65	SUS 5	0.60	BEN 6	0.53
CON 3	0.62	HM 1	0.72	SER 5	0.65	BAR 6	0.59	SUS 4	0.55	BEN 1	0.51
CON 4	0.60	HM 5	0.66	SER 6	0.64	BAR 4	0.58	SUS 1	0.52	BEN 2	0.50
CON 1	0.58	HM 6	0.52	SER 2	0.56	BAR 2	0.56			BEN 5	0.47
CON 6	0.55	HM 7	0.48	SER 7	0.55						
CON 5	0.53										
CON 10	0.53										
CON 9	0.52										
CON 7	0.48										

*SUS* susceptibility, *SER* seriousness, *BEN* benefits, *BAR* barriers, *CON* confidence, *HM* Health motivation

#### Mammography

In determining whether the data is appropriate for EFA, 30 items related to MMG were screened prior to the analysis. The examination of the correlation matrix showed that there are patterned relationships among the variables. In addition, screening exposed the absence of multicollinearity. The observation of Bartlett’s Test of Sphericity (*P* = 0.0001) confirmed that the items have patterned relationships. Finally, the examination of KMO (0.72) verified that the sample size is adequate for EFA. Thus, based on the initial findings, it was demonstrated that factor analysis could be performed on the subscales related to MMG.

The preliminary examination of the eigenvalue displayed eight factors to be extracted, with a total explained variance of 62.65. However, the scree plot displayed six factors to be extracted. Parallel analysis was therefore used in providing further evidence on the decision made on the number of factors. The examination of Table 5 showed that the eigenvalue of the first factor in the actual data is 5.13, whereas, in the simulative data set, it is 2.36. The eigenvalue of the second factor in the actual data is 4.39, while in the simulative data, it is 2.12. The eigenvalue of the third factor in the actual data is 3.47, while in the simulative data, it is 1.95. The eigenvalue of the fourth factor in the actual data is 2.86, while in the simulative data, it is 1.83. The eigenvalue of the fifth factor in the actual data is 2.16, whereas, in the simulative data, it is 1.72. The case concerning the move from the fifth to the sixth factor is different. Accordingly, the number of the scale factors is determined to be limited to five since the eigenvalue of the simulative data of the sixth factor is higher than that of the actual data. The eigenvalue of the sixth factor in the actual data is 1.54, while in the simulative data, it is 1.63. This situation must be regarded as the point where the decision about the number of factors is introduced by parallel analysis.

**Table 5:** Eigen Values of the Actual Data and the Simulative Data

Factor	Eigenvalues of the actual data	Eigenvalues of the simulative data
1	5.13	2.36
2	4.39	2.12
3	3.47	1.95
4	2.86	1.83
5	2.16	1.72
6	1.54	1.63

There is no match between the number of factors attained through the eigenvalue and scree plot methods and those attained from the original scale developmental study, where the number of factors was found to be more than estimated. However, the number of factors obtained through the approach of parallel analysis is parallel to the number of factors obtained from the original scale developmental study [49]. Hence, it was determined that the number of factors is five, and the analysis was re-performed on the actual data, with the restricted number of factors decided by parallel analysis with a total explained variance of 52.63%. The EFA results related to MMG subscales are presented in Table 6.

**Table 6:** Factor analysis results of HBMS for mammogram

Factor 1		Facto 2		Facto 3		Facto 4		Facto 5	
Item	Factor loading	Item	Factor loading	Item	Factor loading	Item	Factor loading	Item	Factor loading
HM 3	0.88	BEN 3	0.76	SER 4	0.76	BAR 3	0.84	SUS 3	0.79
HM 2	0.88	BEN 6	0.74	SER 3	0.69	BAR 4	0.79	SUS 2	0.76
HM 4	0.80	BEN 4	0.73	SER 1	0.67	BAR 2	0.78	SUS 5	0.58
HM 1	0.71	BEN 5	0.73	SER 6	0.67	BAR 1	0.77	SUS 1	0.57
HM 5	0.65	BEN 2	0.69	SER 5	0.66	BAR 5	0.65	SUS 4	0.55
HM 6	0.51	BEN 1	0.66	SER 2	0.55				
HM 7	0.47			SER 7	0.53				

*SUS* susceptibility, *SER* seriousness, *BEN* benefits, *BAR* barriers, *HM* Health motivation

Table 6 shows the results of Varimax rotation; the first factor consists of health motivation items; the second factor consists of the benefits of (MMG) items; the third factor consists of seriousness items; the fourth factor consists of the barriers to (MMG) items, and the fifth factor consists of susceptibility items. Table 6 also shows that the range of the rotated factor loadings in the first factor is between 0.47 and 0.88; those in the second factor is between 0.66 and 0.76; those in the third factor is between 0.53 and 0.76; those in the fourth factor is between 0.65 and 0.84, and the range of the rotated factor loadings of the items in the fifth factor is between 0.54 and 0.79. It is therefore decided that the EFA results for MMG-related subscales are congruent with the results attained in the original scale developmental study conducted by Champion [49].

## Discussion

In this study, the CHBMS for BCS was translated into Arabic language, adapted, and tested for factor structure. This was the first scale of HBM applied to BCS behavior on a sample of Yemeni females living in Klang Valley, Malaysia. The results proved this instrument is a valid and reliable tool to evaluate beliefs on BC and BCS for this population.

There is internal consistency for all scales with a range of between 0.76 and 0.87. This study’s reliability coefficients were in line with the reliability coefficients of the original scale developmental studies conducted by Champion [48, 49], except for the susceptibility and benefits of the BSE scales with lower but acceptable alpha coefficients (0.79 and 0.76), respectively. The Cronbach’s alpha coefficients were also similar to those of Parsa et al. [35], Mohammed et al. [34], Akhtari-Zavare et al. [28], Didarloo et al. [30]. However, the studies conducted by Mikail and Petro-Nustas [33], Dewi [29], Juárez-García et al. [32] reflected a much lower range of alpha coefficients. Therefore, it is recommended for the reliability of these scales to be tested in future studies.

The examination was performed on BSE and MMG items for EFA. All the items in each of the subscales met the loading criteria and were loaded separately on each factor. This has resulted in six factors for BSE and five factors for MMG. In our study, five items were loaded on susceptibility factor, seven items were loaded on seriousness factor, six items were loaded on benefits of BSE factor, six items were loaded on barriers of BSE factor, eleven items were loaded on confidence factor, seven items were loaded on health motivation factor, six items were loaded on benefits of MMG factor, and five items were loaded on barriers of MMG factor. These results are consistent with those obtained in studies conducted by Champion [48, 49], Akhtari-Zavare et al. [28], Didarloo et al. [30], Juárez-García et al. [31], hence validating the scales’ construct validity. However, contrary to our results, Parsa et al. [35] revealed that items regarding Malaysian women’s beliefs about MMG and BSE of BC, and its screening methods were loaded on ten and seven factors, respectively. The results of Juárez-García et al. [32] revealed that items regarding Mexican women’s beliefs about MMG of BC and its screening methods were loaded on six factors. Mikail and Petro-Nustas [33] identified nine factors for BSE. Besides that, nine and six factors were extracted for BSE, and six for MMG, respectively, among Iranian women in Taymoori and Berry [38]. These differences may have resulted from the diverse population, ethnicity, and socio-cultural aspects. The generation gap between the women in the different study groups could be crucial in this regard. Another possibility could be attributed to the insightful teachers who highlighted that they are prepared to gain knowledge on health and spread healthy behaviors compared to other female groups.

All the items in seriousness, susceptibility, barriers of BSE, barriers of MMG, and benefits of MMG factors indicated accepted factor loading, which is congruent with past researches of Champion [49], Mohammed et al. [34], Akhtari-Zavare et al. [28], Didarloo et al. [30], Juárez-García et al. [31]. Thus, the current study's findings supported the theoretical relationships represented in Champion et al. [11], by which there are no significant differences between susceptibility and stages of BCS adoption. Instead, there is a stable level of perceived susceptibility among women who completed the screening and those who have not. The relationship between increased benefits and decreased barriers with BCS compliance is incongruent with the study conducted by Champion et al. [11].

EFA indicated acceptable factor loading for confidence factor loading except for item number seven, “I am able to recognize normal and abnormal breast tissue while performing BSE”, with a low factor loading (0.48) that is close to that obtained by Parsa et al. [35]. However, this was inconsistent with the results of Champion [48], Mikail and Petro-Nustas [33], Akhtari-Zavare et al. [28], Taymoori, and Berry [38]. This observation may have resulted from poor knowledge and cultural relevance of BC and BSE. To identify any abnormal changes in their breasts, women require primary knowledge of the BSE performance method and practice BSE regularly. The BC knowledge and BSE practice rate were found inadequate among Yemeni women [9]. There is a lack of educational interventions for BCS among this group of women [9], which confirms the importance of educating women in the routine and correct examination technique to increase their confidence to conduct BSE correctly.

EFA indicated acceptable factor loading for the health motivation factor. However, three items related to health motivation were unsatisfactory among Yemeni women. Hence, Yemeni, Iranian [30, 38], and Malaysian [35] women's health motivation was very similar. Yemeni women are less likely to receive healthcare benefits, and regular health services are low in the general population. Thus, preventive measures such as “exercising at least three times weekly” and “regular health check-ups” are considered unnecessary among the Yemenis.

Additionally, as foreign immigrants, these women face numerous challenges in accessing health-related information and services. Such challenges include the barriers in language, culture, and health beliefs, the accessibility to transportation, as well as the difficulty in accessing healthcare facilities. Limited healthcare insurance coverage for regular access to health check-ups among most Yemenis in Malaysia adds to the challenge. Furthermore, fatalism and relying on God’s will in illnesses are prevalent among various Arab cultures [16]. Additionally, the family value in Middle Eastern and Muslim cultures makes women dependent on their male counterparts for decision-making of their medical care or their tendency to prioritize family needs before their own health [66]. Therefore, determining the indicators of Yemeni women’s motivation in maintaining and promoting health or in the early detection of diseases requires more refinement and testing of the motivational scale.

The EFA indicated acceptable factor loading for the benefits of BSE factor in this study. However, three items related to this subscale do not seem relevant to this group; “Feeling good about myself when doing BSE”, “When completing monthly BSE, I don’t feel worried as much about BC”, “Completing monthly BSE will decrease the chance of requiring disfiguring or radical surgery if BC occurs”. The study of Didarloo et al. [30] corroborates the present research in this regard. This is possible because most women involved in this study are younger women, who believe there is a higher chance for BC among older women. This may also result from cultural differences and little knowledge on BCS and its benefits. Besides, the prevalence of fatalism and God’s will result in illnesses among various Arab cultures, as stated earlier. A fatalistic attitude would prevent women from comprehending the advantage of early detection [67].

### Strengths of the study

The strengths of the current study include; first, this is the first study to evaluate the reliability and validity of CHBMS on Yemeni women in Malaysia. The second strength is the good response rate. In this study, the dropout rate was low, thus preserving the distribution of the population and assuring the validity of the study results. The study’s final strength is in using the parallel analysis method as a consistent and acceptable method to accurately determine the number of factors.

### Limitation of the study

Despite its strengths, there are some limitations of the study that need to be addressed. First, the limitation of the study population to just one group (schoolteachers). Therefore, the psychometric properties of the CHBMS have been validated only within this group of population. Besides that, due to the small sample size and the restriction of the study location to the Klang Valley area, the findings might not be generalized to all Yemeni women in Malaysia. Consequently, future research is recommended to replicate the results on different groups of women regardless of their workplace, cultures, nationalities, religions, and regions, with a larger sample size to obtain results that can be generalized. Another limitation is the use of self-reporting answer type of questions that may produce a biased judgment. Although inevitable, the researchers have minimized this prejudice by being more objective during the phase of data collection by utilizing simple, precise language and assuring the confidentiality and anonymity of the participants. Apart from that, language and cultural differences are major limitations to adequate BCS in this study since the participants are foreign nationals.

## Conclusions

The translated version of the HBM for evaluating the belief of women regarding BC and BCS was tested for psychometric properties on a sample of Yemeni female teachers living in the Klang Valley, Malaysia; the scales have been found to have acceptable reliability and validity. Healthcare providers could easily use the scales in evaluating the belief of women regarding BC and BCS, which will help them to further promote for BC early detection behavior by planning appropriate educational interventions targeting unsound beliefs and misconceptions. The effectiveness of the intervention programs could also be tested using this instrument. Moreover, this tool could also be used by other Arabic-speaking populations. On the other hand, due to differences in cultural norms amongst the subpopulations, it is thus paramount for these scales to be validated with other Arab women subgroups for norm equivalence, scale, and conceptual aspects. Therefore, to confirm and strengthen the generalization of the study results, the replication and continued psychometric assessment of the Arabic version of Champion’s scales with a similar and diverse population group using a larger sample size is recommended.

## Data Availability

The datasets used and/or analysed during the current study are available from the corresponding author on reasonable request.

## Abbreviations

BC: Breast cancer
BCS: Breast cancer screening
BSE: Breast self‐examination
MMG: Mammography
HBM: Health Belief Model
CHBMS: Champion’s Health Belief Model Scale
EFA: Exploratory factor analysis
KMO: Kaiser–Meyer–Olkin
CVI: Content validity index
